# *LsToll* Gene Mediates Antibacterial Immunity and Developmental Regulation in *Loxostege sticticalis*

**DOI:** 10.3390/insects17060581

**Published:** 2026-06-03

**Authors:** Liqiong Yan, Yasiguleng Bai, Pengwu Zhao, Jianxin Wu, Wenxin Xia, Yanru Zhang, Xiaoli Wang, Liyan Zhang, Haiyan Jiang

**Affiliations:** 1 College of Forestry, Inner Mongolia Agricultural University, Hohhot 010019, China; 15705011843@139.com (Y.B.); zhaopengwu12@163.com (P.Z.); zhangyanru4479@126.com (Y.Z.); nmwangxiaoli@163.com (X.W.); zhangliyanyong@126.com (L.Z.); 2 National Orientation Observation and Research Station of Saihanwula Forest Ecosystem in Inner Mongolia, Chifeng 025000, China; 3 Inner Mongolia Forestry and Grassland General Station, Hohhot 010020, China; 18845780170@163.com; 4 Dandong Forestry and Grassland Discovery Service Center, Dandong 118000, China; 18341048004@163.com

**Keywords:** *Loxostege sticticalis*, Toll receptor gene, innate immunity, RNA interference

## Abstract

Toll-like receptors (TLRs) are conserved pattern recognition receptors essential to insect innate immunity. However, the functions of TLRs in *Loxostege sticticalis*, a destructive agricultural pest, remain poorly characterized. In this study, the full-length coding sequence of the *L. sticticalis* Toll receptor (*LsToll*) was identified and characterized to analyze its molecular features. Structural analysis showed that *LsToll* possesses typical Toll family features, including an extracellular domain containing 19 leucine-rich repeats (LRRs), a transmembrane helix, and a highly conserved intracellular Toll/interleukin-1 receptor (TIR) domain. *LsToll* transcript levels were significantly upregulated after bacterial challenge. RNAi-mediated silencing of *LsToll* significantly reduced larval tolerance to bacterial infection and increased mortality. Notably, *LsToll* suppression also induced severe developmental abnormalities, including molting obstruction, pupation failure, and defects in wing expansion in newly emerged adults. Transcriptome analysis after RNAi identified 5230 differentially expressed genes (DEGs), which were significantly enriched in insect hormone biosynthesis and metabolic pathways. Biochemical assays further confirmed that *LsToll* knockdown decreased 20-hydroxyecdysone (20E) titers and increased juvenile hormone III (JH III) titers. These results suggest that *LsToll* contributes to antibacterial defense and normal development in *L. sticticalis.* Its involvement in both survival and development indicates that *LsToll* may serve as a promising molecular target for sustainable pest management strategies.

## Simple Summary

The beet webworm, *Loxostege sticticalis*, is a destructive migratory pest. Long-term reliance on chemical control has led to increasingly serious problems, including insecticide resistance and environmental pollution. In this study, we identified and cloned a Toll-like receptor gene, *LsToll*, from *L. sticticalis*. *LsToll* expression was significantly induced under bacterial challenge. RNA interference (RNAi)-mediated suppression of *LsToll* reduced the antibacterial capacity of larvae and increased mortality. Moreover, *LsToll* silencing caused severe developmental defects, including molting obstruction, pupation failure, and wing deformities in newly emerged adults. Transcriptome analysis identified differentially expressed genes (DEGs) following *LsToll* knockdown, which were significantly enriched in metabolic pathways and insect hormone biosynthesis pathways. Biochemical assays further showed that *LsToll* silencing decreased the 20-hydroxyecdysone (20E) titer and increased the juvenile hormone III (JH III) titer. This research elucidates the dual roles of *LsToll* in immunity and development, providing new insights into Toll receptor genes as potential targets for sustainable pest management.

## 1. Introduction

Insects rely exclusively on innate immunity, as they lack the adaptive immune system characteristic of vertebrates. Innate immunity plays a fundamental role in protecting insects from diverse pathogenic challenges through the coordinated action of cellular and humoral responses [1]. Cellular immunity is primarily mediated by hemocytes, which eliminate pathogens through processes such as phagocytosis, encapsulation, and nodulation [2]. In contrast, humoral immunity is initiated by the recognition of pathogen-associated molecular patterns (PAMPs) by pattern recognition receptors (PRRs), leading to the induction of antimicrobial peptides (AMPs) in the hemolymph. This process is mainly regulated by conserved immune signaling pathways, including Toll, immune deficiency (IMD), and Janus kinase/signal transducer and activator of transcription (JAK/STAT) pathways [3].

The Toll pathway is an evolutionarily conserved innate immune pathway that plays a pivotal role in insect defense against fungal, bacterial, and viral pathogens [4,5]. This pathway is centered on transmembrane Toll-like receptors (TLRs), a conserved family of pattern recognition receptors. These receptors contain extracellular leucine-rich repeat (LRR) domains involved in ligand recognition and intracellular Toll/interleukin-1 receptor (TIR) domains that mediate downstream signal transduction [6]. The first Toll receptor, Toll1, was identified in the dipteran *Drosophila melanogaster*, where it was shown to be indispensable for both embryonic dorsoventral patterning and immune responses [7,8]. Subsequent studies identified TLRs in mammals, demonstrating that Toll/TLR-mediated signaling is deeply conserved; however, receptor activation mechanisms differ substantially between insects and mammals, reflecting lineage-specific functional specialization [9,10]. Unlike mammalian TLRs, which directly bind microbial PAMPs, insect Toll receptors (Tolls) are generally activated indirectly through the cysteine-knot cytokine Spätzle, which undergoes proteolytic cleavage upon infection to become a functional ligand [11]. Beyond pathogen defense, Tolls are also involved in diverse physiological processes, including AMP production, phagosome regulation, and inflammatory signaling [12]. In the dipteran *Anopheles gambiae*, four *Toll* genes have been characterized, among which *AgToll9* is notably upregulated following challenge with *Escherichia coli* [13]. In lepidoptera, Toll signaling has been extensively studied in the lepidopteran *Bombyx mori*, in which fourteen *Toll* genes have been identified [14]. Functional studies revealed that overexpression of *BmToll9-1* in Bm5 cells upregulates *Dicer-2*, a core component of the RNA interference (RNAi) machinery. Under lipopolysaccharide (LPS) stimulation, *BmToll9-1* overexpression suppresses the expression of AMPs and immune effector genes associated with the IMD and JAK/STAT pathways. Furthermore, RNAi-mediated silencing of *BmToll9-2* significantly impairs larval growth and body weight, and this phenotype can be reversed after bacterial infection when *BmToll9-2* expression is restored. These observations suggest that *Toll* genes can contribute to both innate immunity and growth regulation [15].

The beet webworm, *Loxostege sticticalis* (Lepidoptera: Pyralidae), is a destructive migratory pest that inflicts severe ecological and economic damage across North America, Eastern Europe, and Asia [16,17]. As an extensively polyphagous species, *L. sticticalis* feeds on over 200 plant species spanning 35 families, posing a substantial threat to global crop production [18,19]. Current research on *L. sticticalis* has primarily focused on migratory behavior, outbreak forecasting, and the effects of environmental stressors on development and reproduction [20,21,22,23]. With the recent completion of a chromosome-level genome assembly for this species, research has progressively expanded from ecological and physiological studies to molecular investigations, particularly in relation to olfactory perception and stress resistance [24,25,26]. Despite these advances, the immunobiology of *L. sticticalis* remains poorly understood, and existing studies are limited mainly to the preliminary characterization of individual genes, such as lysozyme [16]. Consequently, the molecular mechanisms governing the innate immune signaling network in *L. sticticalis* have not been systematically elucidated. Targeting innate immune signaling may provide a strategy for biological pest control, as suppression of key immune genes can increase insect susceptibility to pathogens and enhance the efficacy of microbial control agents [27,28].

In the present study, a Toll receptor gene from *L. sticticalis*, designated *LsToll*, was identified and characterized using publicly available genomic and transcriptomic datasets (BioProject number: PRJNA1118492) [29]. The expression profiles of *LsToll* across different developmental stages and tissues, as well as its transcriptional response to bacterial challenge, were quantified using real-time quantitative PCR (RT-qPCR). The functional involvement of *LsToll* in growth, development, and immune defense was further evaluated through RNAi-mediated knockdown. In addition, RNA sequencing (RNA-seq), RT-qPCR validation of selected differentially expressed genes (DEGs), and hormone titer measurements were performed to explore downstream changes associated with *LsToll* silencing. Together, these analyses provide insight into the roles of *LsToll* in antibacterial immunity and developmental regulation in *L. sticticalis*.

## 2. Materials and Methods

### 2.1. Insect Strains, Rearing, and Sample Collection

In June 2025, more than 1000 second- to fifth-instar larvae of *L. sticticalis* were collected from Hohhot, Inner Mongolia, China (40°82′17″ N, 111°71′58″ E). The larvae were reared on fresh *Chenopodium album* under controlled laboratory conditions at 22 ± 1 °C, 75 ± 5% relative humidity, and a photoperiod of 16 h light:8 h dark (L:D). Last-instar larvae were transferred to plastic containers containing clean sandy soil maintained at approximately 15% humidity to facilitate pupation. Newly emerged adults were supplied with a 5% honey solution and allowed to oviposit on young *C. album* plants.

To analyze the expression profiles of *LsToll* at different developmental stages, eggs, larvae from the first to fifth instars, male and female pupae, and male and female adults of *L. sticticalis* were collected. For tissue-specific expression analysis, day-1 fifth-instar larvae were dissected to obtain the head, integument, fat body, foregut, midgut, hindgut, Malpighian tubules, and hemolymph. All tissues were dissected in cold phosphate-buffered saline (PBS, pH 7.4), immediately frozen in liquid nitrogen, and stored at −80 °C until RNA extraction. Each treatment included three biological replicates, with each replicate containing at least three individuals.

### 2.2. Bacterial Challenge

*Staphylococcus aureus* and *Escherichia coli* were purchased from InvivoGen (San Diego, CA, USA). Both strains were cultured overnight in Luria–Bertani broth at 37 °C with shaking at 200 rpm and then subcultured to the mid-logarithmic phase (OD_600_ = 0.5). Bacterial cells were collected by centrifugation, washed twice with sterile PBS, and resuspended in PBS to a final concentration of approximately 1.0 × 10^7^ cells/mL.

Newly molted third-instar larvae were starved for 24 h and then individually fed standardized leaf disks coated with 10 μL of bacterial suspension. Control larvae were fed leaf disks treated with sterile PBS. The larvae were maintained under standard rearing conditions. Each treatment included three biological replicates, with 20 larvae per replicate. Whole larvae were collected at 6, 12, 24, and 48 h post-feeding, rapidly frozen in liquid nitrogen, and stored at −80 °C before RNA extraction.

### 2.3. RNA Interference (RNAi) and Biological Assays

Based on the coding sequence of *LsToll*, four siRNAs (si*LsToll-888*, si*LsToll-946*, si*LsToll-1298*, and si*LsToll-1695*) and a negative control siRNA (si*NC*) were designed using the siRNA design service provided by Cenix BioScience (Cenix BioScience GmbH, Dresden, Germany) and synthesized by GenePharma (Shanghai GenePharma Co., Ltd., Shanghai, China) (Table A1). Fourth-instar larvae were microinjected with 1 μL of siRNA (500 ng/μL) into the penultimate abdominal segment using a MICROLITER™ 65 microinjector equipped with a 33-gauge needle (Hamilton, Reno, NV, USA) under ice anesthesia. The larvae were subsequently reared under standard conditions. Larvae were collected at 24, 48, and 72 h post-injection for RNA extraction. Each treatment included three biological replicates, with 30 larvae per replicate. The most effective siRNA was selected based on gene-silencing efficiency determined by RT-qPCR.

For growth and developmental assays, fourth-instar larvae injected with si*LsToll* or si*NC* were monitored daily for survival, molting, and pupation for 10 days after siRNA injection. Fifty larvae were used in each group to assess survival rates. For antibacterial assays, thirty healthy larvae at 24 h post-injection were randomly selected from each group and subjected to bacterial challenge as described in Section 2.2. Following bacterial challenge, larval mortality was recorded at 24 h intervals for 10 days.

### 2.4. Transcriptome Sequencing and Analysis

Based on the RNAi efficiency assessment, the lowest *LsToll* transcript levels were observed at 48 h post-injection. Accordingly, larvae from the si*LsToll* and si*NC* groups were collected at this time point for transcriptome sequencing. Three independent biological replicates were prepared for each group, and each replicate consisting of three larvae. Transcriptome sequencing was performed by Novogene (Novogene, Beijing, China). Raw reads were filtered to obtain high-quality clean reads, and quality metrics, including Q20, Q30, and GC content were assessed. Clean reads were aligned to the *L. sticticalis* reference genome using HISAT2 (v0.6.1). Transcript assembly was performed using Trinity, and unigenes were obtained after removing redundant sequences. Functional annotation was conducted by comparison with the Swiss-Prot, Gene Ontology (GO), Kyoto Encyclopedia of Genes and Genomes (KEGG), and Pfam databases. DEGs between the si*LsToll* and si*NC* groups were identified using the DESeq package (v1.18.0) in R software (v4.6.0). *p*-values were adjusted using the Benjamini–Hochberg method, and genes with |log_2_(fold change)| ≥ 1 and False Discovery Rate (FDR) ≤ 0.05 were considered significantly differentially expressed. KEGG enrichment analyses were performed using clusterProfiler (v8.1), with FDR ≤ 0.05 as the significance threshold. To validate the transcriptome results, five DEGs were selected for RT-qPCR analysis. The RNA samples used for RT-qPCR were identical to those used for transcriptome sequencing (Table A1).

### 2.5. Determination of 20E and JH III Titers

To determine whether *LsToll* silencing altered endocrine hormone homeostasis, the titers of 20-hydroxyecdysone (20E) and juvenile hormone III (JH III) were measured in larvae from the si*LsToll* and si*NC* groups at 48 h post-injection. The levels of 20E and JH III were determined using commercial ELISA kits (Gelatins, Shanghai, China) according to the manufacturer’s instruction [30]. Briefly, larvae were collected on ice after RNAi treatment, homogenized in PBS (pH 7.2–7.4), and centrifuged at 5000× *g*. The supernatants were collected for ELISA analysis. Standards and samples were added to hormone antibody-coated microplates, followed by HRP-labeled detection antibodies. After washing, 3,3′,5,5′-tetramethylbenzidine (TMB) substrate was added for color development, and absorbance (OD value) was measured at 450 nm using a microplate reader. Hormone titers were calculated according to the corresponding standard curves. Each treatment included three biological replicates, and each sample was measured with three technical replicates.

### 2.6. Statistical Analysis

All quantitative data are presented as the mean ± standard error of the mean (SEM). Prior to statistical analysis, data were assessed for normality using the Shapiro–Wilk test and for homogeneity of variance using Levene’s test. Data that did not meet the assumptions of normality or homogeneity of variance were log-transformed before further analysis. Comparisons between two groups were performed using an unpaired Student’s *t*-test, whereas comparisons among multiple groups were conducted using one-way analysis of variance (ANOVA) followed by Tukey’s multiple-comparison test. Survival data were analyzed using the Kaplan–Meier method, and differences between groups were assessed using the log-rank test. All statistical analyses were performed using SPSS version 25.0 (IBM Corp., Chicago, IL, USA), and figures were generated using GraphPad Prism 8.3 (GraphPad Software Inc., La Jolla, CA, USA). Differences were considered statistically significant at *p* < 0.05.

## 3. Results

### 3.1. Sequence Analysis of LsToll

**Figure 1:**
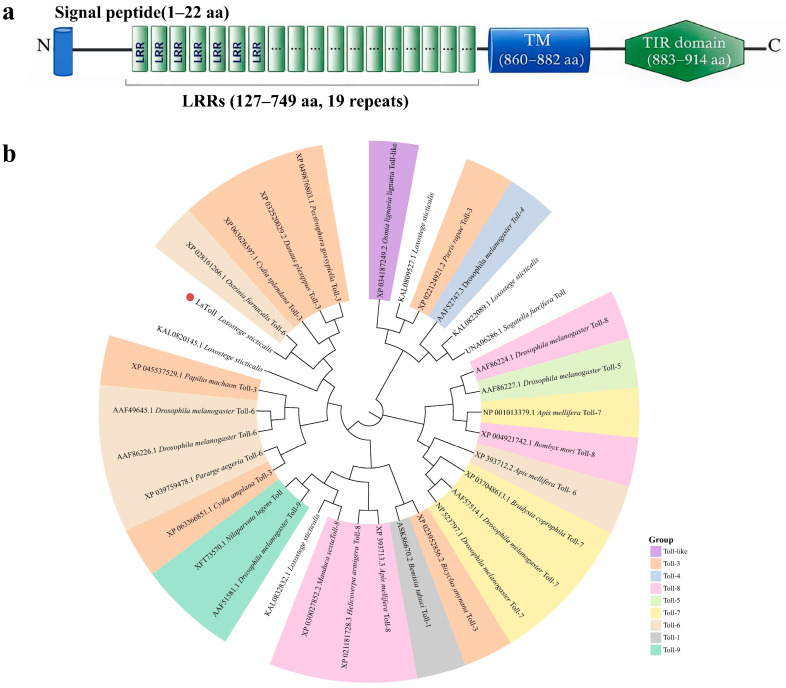
Sequence analysis of LsToll. (**a**) Domain organization of LsToll predicted by the SMART program. LRR, leucine-rich repeat domain; TM, transmembrane domain; TIR, Toll/interleukin-1 receptor domain. (**b**) Phylogenetic tree of LsToll and other insect Toll proteins constructed from protein sequences using the neighbor-joining method. Numbers at the nodes indicate bootstrap support values (%) from 1000 replicates. GenBank accession numbers are provided in parentheses. The red dot represents the protein of the target species in this study.

The full-length coding sequence of *LsToll* (GenBank accession no. KAL0820158.1) was identified and comprises 2745 bp, encoding a protein of 915 amino acids with a predicted molecular weight of 103.6 kDa and an isoelectric point of 6.75. Structural analysis revealed that LsToll exhibits the characteristic features of a TLR, including an N-terminal signal peptide (amino acids 1–22), an extracellular region containing 19 LRR motifs (amino acids 127–749), a single transmembrane helix (860–882 aa), and a conserved C–terminal TIR domain (883–914 aa) (Figure 1a).

The phylogenetic tree was constructed using five *Toll* sequences from *L. sticticalis* (*LsToll*, KAL0809527.1, KAL0820145.1, KAL0822089.1, and KAL0832832.1), together with homologous Toll protein sequences from Lepidoptera, Diptera, Hymenoptera, and other insect groups. The phylogenetic results showed that these *L. sticticalis* Toll proteins did not form a single monophyletic clade but were distributed across different evolutionary branches. Notably, LsToll was clustered most closely with Toll6 from lepidopteran *Ostrinia furnacalis* (Figure 1b).

### 3.2. Developmental and Tissue-Specific Expression Profiles of LsToll

**Figure 2:**
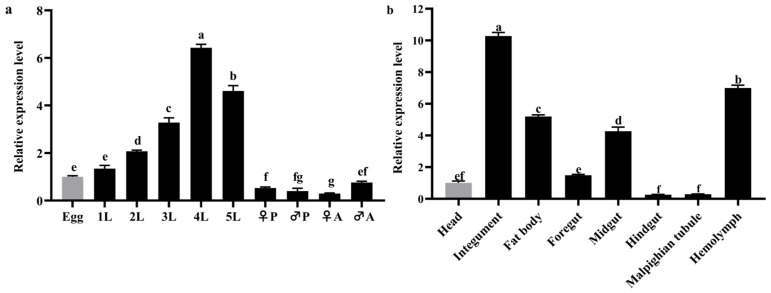
Developmental and tissue-specific expression profiles of *LsToll*. (**a**) Developmental expression levels of the *LsToll* in *L. sticticalis*. Developmental stages: 1–5 L, first- to fifth-instar larvae; ♀P, female pupae; ♂P male pupae; ♀A, female adults; ♂A, male adults. (**b**) Tissue-specific expression levels of *LsToll* in fifth-instar larvae of *L. sticticalis*. Data are expressed as mean ± SEM (*n* = 3). Different lowercase letters above the columns indicate significant differences at *p* < 0.05.

The developmental and tissue-specific expression patterns of *LsToll* in *L. sticticalis* were quantified by RT-qPCR (Figure 2). Transcripts of *LsToll* were detected at all examined developmental stages, including eggs, larvae, pupae, and adults, indicating constitutive expression throughout the life cycle. Notably, the expression of *LsToll* was significantly elevated in fourth- and fifth-instar larvae, showing 7.62- and 4.65-fold increases, respectively, compared with the egg stage (Figure 2a).

Tissue-specific expression analysis in fifth-instar larvae showed that *LsToll* was expressed in all tested tissues, including the head, integument, fat body, foregut, midgut, hindgut, Malpighian tubules, and hemolymph. The highest transcript level was detected in the integument, which was 10.17-fold higher than that in the head, followed by the hemolymph, where expression was 8.75-fold higher than that in the head. In contrast, relatively low expression levels were observed in the hindgut and Malpighian tubules, corresponding to 0.31- and 0.37-fold of the expression level in the head, respectively (Figure 2b).

### 3.3. Transcriptional Response of Toll Signaling Pathway Genes Following Oral Bacterial Challenge

**Figure 3:**
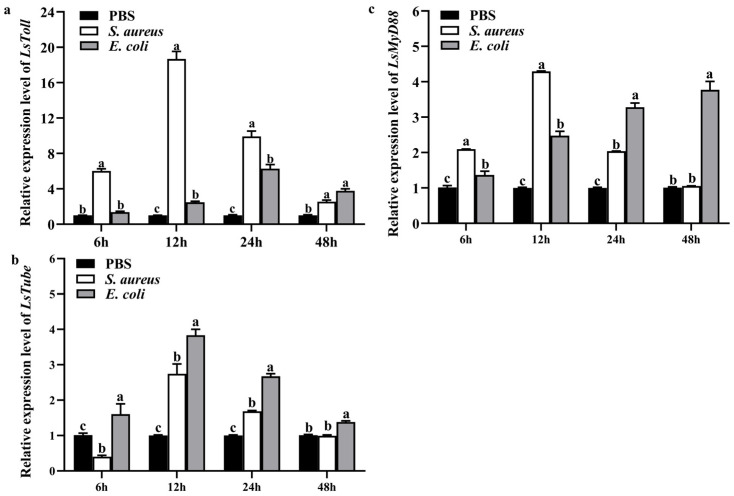
Whole-body expression profiles of Toll signaling pathway genes after oral bacterial challenge. (**a**) *LsToll* (**b**) *LsMyD88* (**c**) *LsTube* expression of genes following *S. aureus* and *E. coli* challenges in fourth-instar *L. sticticalis* larvae. Data are expressed as mean ± SEM (*n* = 3). Different lowercase letters above the columns indicate significant differences at *p* < 0.05.

The temporal expression patterns of *LsToll*, *LsMyD88*, and *LsTube* following oral bacterial challenge were examined by RT-qPCR after exposure to *S. aureus* and *E. coli* (Figure 3). Overall, the three genes showed distinct but inducible responses to bacterial stimulation. After *S. aureus* challenge, *LsToll* was rapidly and strongly upregulated, increasing 6.03-fold at 6 h and peaking at 18.62-fold at 12 h. *LsMyD88* showed a similar early response pattern, with expression increasing to approximately 2.10-fold at 6 h and peaking at 4.29-fold at 12 h. In contrast, *LsTube* was initially downregulated at 6 h but was subsequently induced, reaching its highest level of approximately 2.74-fold at 12 h.

Following *E. coli* challenge, the induction patterns of these genes were generally delayed compared with those induced by *S. aureus*. *LsToll* showed moderate induction at early time points and peaked at 24 h, reaching 9.93-fold relative to the control. *LsMyD88* exhibited a sustained increase after *E. coli* challenge, rising from approximately 1.37-fold at 6 h to 3.77-fold at 48 h. *LsTube* was also induced by *E. coli*, with expression peaking at approximately 3.83-fold at 12 h and remaining elevated at 24 h.

### 3.4. Optimization of siRNA Selection for LsToll

**Figure 4:**
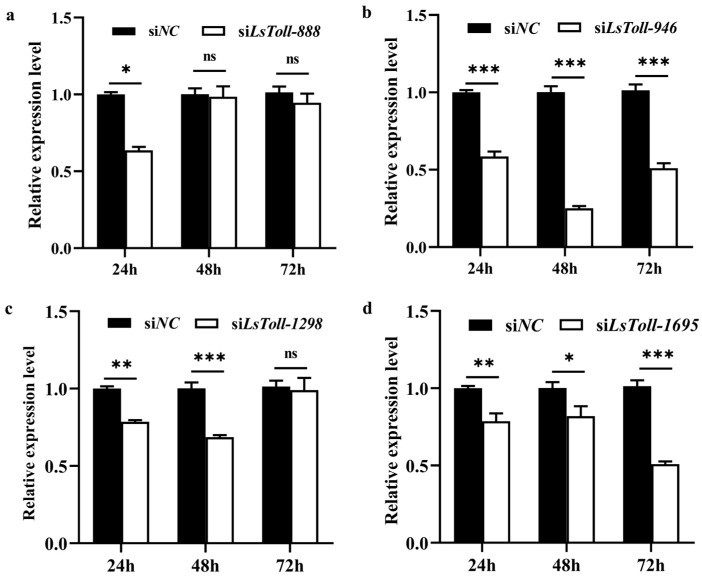
Efficiency of siRNA-mediated silencing of *LsToll*. (**a**) si*LsToll-71*, (**b**) si*LsToll-888*, (**c**) si*LsToll-1298*, (**d**) si*LsToll-1695*. Data are expressed as mean ± SEM (*n* = 3). Statistical significance was determined by the unpaired Student’s *t*-test (*, *p* < 0.05; **, *p* < 0.01; ***, *p* < 0.001). Values marked with “ns” are not significantly different (*p* > 0.05).

Four siRNAs targeting *LsToll* (si*LsToll-888*, si*LsToll-946*, si*LsToll-1298*, and si*LsToll-1695*) were synthesized and injected into fourth-instar larvae of *L. sticticalis* to evaluate gene-silencing efficiencies. Injection of si*LsToll-888* resulted in a significant reduction in *LsToll* expression only at 24 h post-injection, reaching 0.64-fold of the control level, whereas no significant silencing was detected at 48 or 72 h (Figure 4a). In contrast, si*LsToll-946* significantly reduced *LsToll* expression at 24, 48, and 72 h post-injection, with transcript levels decreasing to 0.58-, 0.25-, and 0.51-fold of the control level, respectively (Figure 4b). For si*LsToll-1298*, moderate silencing was observed at 24 h (0.79-fold) and 48 h (0.69-fold), but no significant reduction was detected at 72 h (Figure 4c). Injection of si*LsToll-1695* resulted in significant suppression of *LsToll* expression at all three time points, with transcript levels decreasing to 0.79-, 0.82-, and 0.51-fold at 24, 48, and 72 h, respectively (Figure 4d). Based on its consistent silencing efficiency, si*LsToll-946* was selected for subsequent functional analyses.

### 3.5. Functions of LsToll Revealed by RNAi

**Figure 5:**
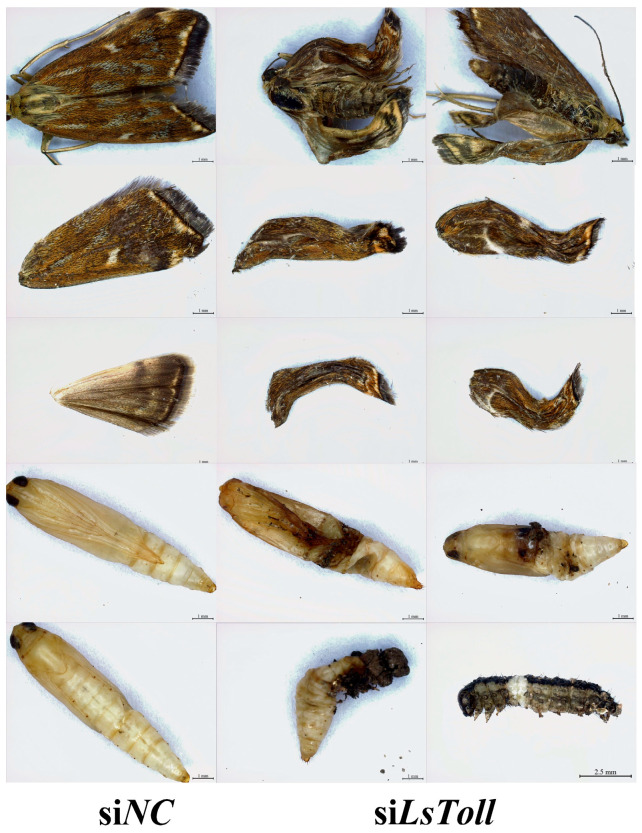
Abnormal phenotypes resulting from the silencing of *LsToll* mediated by RNAi.

Compared with larvae injected with si*NC*, *LsToll* silencing significantly reduced both pupation and survival rates, with pupation decreasing to 58% and survival decreasing to 34% (Table 1). Furthermore, larvae exhibited pronounced developmental abnormalities during the prepupal stage, including incomplete pupation and malformed pupal cases, ultimately leading to death (Figure 5). The malformation rate was 5.56% in the si*NC* group, whereas it increased significantly to 31.81% in the si*LsToll* group (Table 1). In addition, adults emerging from the si*LsToll* group displayed defects in wing expansion, including wing curling and folding (Figure 5). The effects of *LsToll* silencing on the expression of the Toll pathway genes *LsMyD88* and *LsTube* are shown in Supplementary Figure S1.

**Figure 6:**
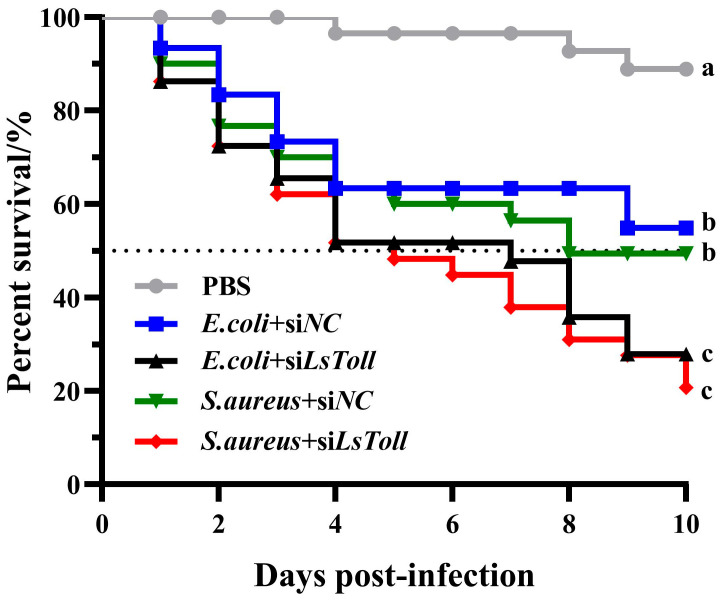
Effects of *LsToll* silencing on the survival of *L. sticticalis* larvae following bacterial challenge. Fourth-instar larvae injected with si*LsToll* or si*NC* were challenged with *E. coli* or *S. aureus*, and larval survival was recorded daily for 10 days post-infection. Larvae treated with PBS served as the control group. Survival curves were generated using the Kaplan–Meier method, and differences among groups were analyzed using the log-rank test followed by Holm correction for multiple comparisons. Different lowercase letters adjacent to the endpoints of the survival curves indicate significant differences among groups (*p* < 0.05), whereas groups sharing the same letter are not significantly different. The dotted line in the survival curve represents the 50% survival rate reference line.

To assess the role of *LsToll* in antibacterial defense, larval survival was examined following bacterial challenge after RNAi treatment. Larvae injected with si*LsToll* showed significantly reduced survival compared with the corresponding *NC* groups after infection with either *E*. *coli* or *S. aureus* (Figure 6). In the absence of infection, mortality in the *NC* group was low (10.0%). Following challenge with *E. coli*, mortality increased to 43.33% in the *NC* group and further rose to 66.67% in the larvae with *LsToll* silenced. A similar pattern was observed after infection with *S. aureus*, with mortality reaching 50.00% in the *NC* group and increasing to 76.67% in the larvae treated with si*LsToll*.

### 3.6. DEGs Analysis Following LsToll RNAi

**Figure 7:**
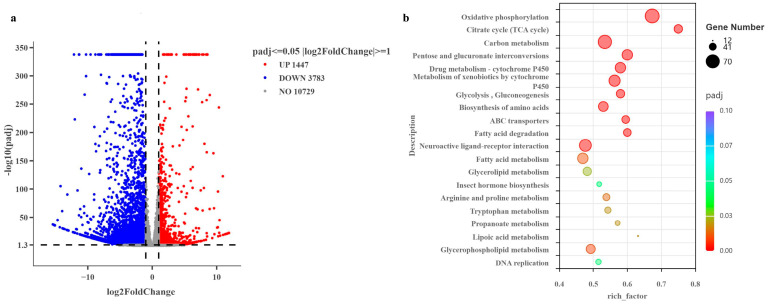
Transcriptomic analysis of *LsToll*-silenced larvae. (**a**) Volcano plot of DEGs. Each dot represents a gene: gray dots indicate genes with no significant difference; red dots indicate significantly upregulated genes; blue dots indicate significantly downregulated genes (|log_2_FoldChange| ≥ 1, *p*adj ≤ 0.05). (**b**) KEGG pathway enrichment analysis of DEGs. Bubble size corresponds to the number of genes in each pathway, and color reflects adjusted *p*adj.

A total of 5230 DEGs were identified, including 1447 upregulated and 3783 downregulated genes (Figure 7a). KEGG pathway enrichment analysis revealed that these DEGs were primarily associated with metabolic and oxidative stress-related pathways, particularly those involved in carbon and fatty acid metabolism. Notably, the insect hormone biosynthesis pathway was significantly enriched, and multiple genes involved in 20E and JH signaling exhibited marked expression changes (Figure 7b).

### 3.7. Changes in 20E and JH III Titers and Validation of DEGs After LsToll Silencing

**Figure 8:**
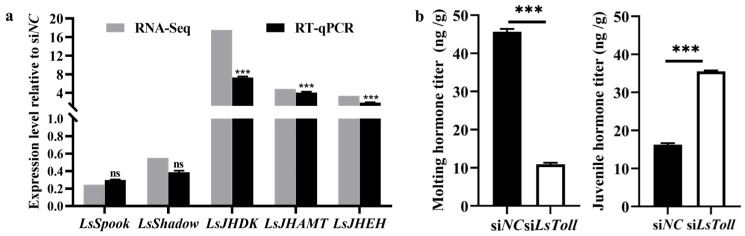
Effects of *LsToll* silencing on hormone-related gene expression and endocrine hormone titers in *L. sticticalis* larvae. (**a**) RT-qPCR validation of DEGs. Two 20E pathway-related genes, *LsSpook* and *LsShadow*, were downregulated after *LsToll* knockdown, whereas three JH pathway-related genes, *LsJHAMT*, *LsJHDK*, and *LsJHEH*, were upregulated. The expression trends detected by RT-qPCR were consistent with the RNA-seq data. (**b**) Changes in 20E and JH III titers at 48 h after siRNA injection. *LsToll* knockdown significantly reduced the 20E titer and increased the JH III titer compared with the si*NC* group. Data are expressed as mean ± SEM (*n* = 3). Significant differences were calculated using an unpaired Student’s *t*-test (***, *p* < 0.001). Values marked with “ns” are not significantly different (*p* > 0.05).

The RT-qPCR results showed expression trends consistent with those observed in the RNA-seq analysis. In RNA-seq dataset, the normalized expression levels of *LsSpook* and *LsShadow* in si*LsToll* group were 0.24- and 0.55-fold of those in si*NC* group, respectively. Consistently, RT-qPCR analysis showed that their relative expression levels were approximately 0.30- and 0.39-fold of those in si*NC* group, respectively. In contrast, *LsJHAMT*, *LsJHDK*, and *LsJHEH* were upregulated after *LsToll* silencing. Their normalized expression levels in RNA-seq dataset were 4.86-, 17.55-, and 3.32-fold of those in si*NC* group, respectively, while RT-qPCR analysis showed corresponding relative expression levels of approximately 4.04-, 7.29-, and 1.88-fold of those in si*NC* group, respectively (Figure 8a). Although the magnitude of the expression changes detected by RT-qPCR differed from that observed in RNA-seq data, the overall expression trends were consistent, supporting the reliability of transcriptomic analysis.

Compared with the si*NC* group, *LsToll* knockdown significantly decreased the 20E titer by approximately 34.72% (Figure 8b). In contrast, JH III exhibited the opposite trend; its titer was significantly elevated in the si*LsToll* group by approximately 2.18-fold relative to the control (Figure 8b).

## 4. Discussion

TLRs serve as pivotal PRRs within the innate immune system [31]. Since the discovery of *Toll1* in *D. melanogaster*, multiple *Toll* genes have been identified in diverse insect species, including the *A. gambiae* [32], the hymenopteran *Apis mellifera* [33], the coleopterans *Tribolium castaneum* [34], *Tenebrio molitor* [35], and *Leptiontarsa decemlineata* [36,37]. In the present study, a *Toll* gene was identified in *L. sticticalis*. Sequence analysis confirmed that *LsToll* is a typical member of the TLR superfamily, characterized by conserved domain [38]. Structurally, *LsToll* is predicted to be a single-pass type I transmembrane protein, containing an extracellular domain with multiple LRRs, a single transmembrane helix, and a conserved C-terminal TIR domain. The LRR region may contribute to ligand recognition, whereas the TIR domain is essential for downstream signal transduction through the recruitment of cytosolic adaptor proteins [38]. Furthermore, phylogenetic analysis demonstrated that LsToll clusters closely with Lepidopteran Tolls, particularly Toll6 from *O. furnacalis*, suggesting that this gene is evolutionarily conserved within Lepidoptera.

In the present study, *LsToll* was expressed throughout all examined developmental stages, a pattern consistent with *NlToll1* in the hemipteran *Nilaparvata lugens* and *AgToll/AgToll9* in *A. gambiae* [13,39]. The elevated *LsToll* transcript levels in fourth- and fifth-instar larvae are noteworthy because these stages involve rapid biomass accumulation and physiological preparation for metamorphosis [40]. This stage-specific expression suggests that *LsToll* may be associated with developmental processes in addition to its potential role in innate immunity. Tissue-specific expression analysis revealed that *LsToll* was predominantly expressed in the integument, hemolymph, fat body, and midgut. These tissues are important immune-related sites in insects and are involved in pathogen recognition, AMP synthesis, barrier defense, and systemic immune regulation [41,42]. While expression levels were comparatively lower in the hindgut and Malpighian tubules, the overall tissue distribution aligns with patterns observed in other species. For instance, *NlToll1* is highly expressed in the fat body and gut of *N. lugens* [39]. In *T. molitor*, *TmToll8* and *TmToll9* are predominantly expressed in the gut, whereas *TmToll10* is enriched in hemocytes [24]. Similarly, *BmToll9* in *B. mori* is enriched in the gut but is barely detectable in specialized tissues such as the silk glands [43].

To clarify the immune function of *LsToll*, its transcriptional profile was analyzed under oral bacterial challenge. *LsToll* was significantly induced by both *S. aureus* and *E. coli*, supporting its involvement in antibacterial immune responses. Similar inducible expression patterns have been reported in other insects. In *B. mori*, BmToll9-2 is responsive to bacterial stimulation, particularly to *E. coli* and its cell wall component LPS [44]. In addition, *BtToll* is induced by both *E. coli* and *S. aureus* in the hemipteran *Bemisia tabaci* [45], whereas *MsToll* shows hemocyte-specific upregulation after *E. coli* challenge in lepidopteran *Manduca sexta* [46]. Notably, *LsToll* showed a stronger and earlier response to *S. aureus* than to *E. coli*, whereas the peak induction after *E. coli* treatment occurred later. This difference may reflect distinct PAMP exposure patterns and upstream immune-recognition mechanisms between Gram-positive and Gram-negative bacteria. Gram-positive bacterial cell walls are generally enriched in lysine (Lys)-type peptidoglycan, which can be recognized by upstream recognition molecules such as PGRP-SA and PGRP-SD, thereby activating the Spätzle-dependent Toll signaling cascade [47]. In contrast, Gram-negative bacteria mainly contain meso-diaminopimelic acid (DAP)-type peptidoglycan, whose recognition is usually associated with activation of the IMD pathway [48]. Therefore, the early immune response induced by *E. coli* may be primarily reflected in IMD-related genes or effectors rather than in rapid *LsToll* transcriptional induction [49]. Activation of the Toll pathway by *E. coli* may occur at later stages of infection, particularly during bacterial persistence or under higher immune pressure. This may explain why the *LsToll* expression peak after *E. coli* treatment appeared later than that after *S. aureus* treatment.

Increasing evidence indicates that *Toll* genes can also contribute to insect growth and development [50]. The inhibition of *NlToll1* in *N*. *lugens* leads to a significant reduction in nymphal survival rates [41], while interference with *LgToll* in *Leguminivora glycinivorella* hinders normal metamorphosis from the larval to pupal stage [51]. Similarly, ingestion of dsRNA targeting *Toll6* results in high mortality in thysanopteran *Frankliniella occidentalis* [52]. In the present study, *LsToll* silencing interfered with normal development in *L. sticticalis*, affecting larval survival, pupation, and adult emergence. The observed defects, including molting obstruction, incomplete pupation, malformed pupae, and abnormal wing expansion, suggest that *LsToll* is associated with developmental regulation. However, these phenotypes should be interpreted as evidence of functional involvement rather than proof that *LsToll* alone controls these developmental transitions.

In insects, 20E and JH are two major endocrine signals that coordinate molting and metamorphosis. 20E promotes molting and stage transitions, whereas JH helps maintain larval characteristics and modulates the developmental outcome of 20E signaling in a stage-dependent manner [53,54]. In this study, *LsToll* knockdown was associated with reduced 20E levels and elevated JH III levels. These hormone changes may provide a plausible physiological explanation for the observed molting obstruction, pupation failure, and adult wing expansion defects. Consistently, transcriptomic and RT-qPCR analyses showed that *LsSpook* and *LsShadow*, two genes associated with the 20E biosynthetic pathway, were downregulated after *LsToll* silencing, whereas *LsJHAMT*, *LsJHDK*, and *LsJHEH*, genes related to JH biosynthesis or metabolism, were upregulated. Because JH-related genes include both biosynthetic and degradation-associated components, these changes should be interpreted as a disruption of JH homeostasis rather than simple activation of JH biosynthesis. Previous studies have shown extensive interactions between endocrine hormones and insect immunity. Much of the current evidence focuses on how hormonal signals regulate immune responses, including the effects of 20E and JH on AMP expression and immune competence in *D. melanogaster*, *A. gambiae*, *A. aegypti*, and *B. mori* [55,56,57,58]. The present study provides evidence that knockdown of an immune-related Toll gene is accompanied by altered expression of hormone-related genes and changes in hormone titers. These results provide a basis for further investigation of Toll-mediated immune–endocrine interactions and may help identify molecular targets for sustainable pest management. Nevertheless, these results do not yet demonstrate that *LsToll* directly regulates 20E or JH biosynthesis. Further studies, including hormone rescue assays and promoter-level regulatory analyses, are needed to clarify the underlying regulatory mechanisms.

## Figures and Tables

**Table 1:** The phenotype and survival rate of *L. sticticalis* microinjected by si*LsToll*.

Treatment	Number of Larvae	Pupation Rate% (Pupae)	Aberration Rate of Pupae% (Individual)	Survival Rate% (Individual)	Aberration Rate of Adults% (Individual)
si*NC*	50	76% (38)	5.56% (2)	70% (35)	0 (0)
si*LsToll*	50	58% (29)	31.81% (7)	34% (17)	35.30% (6)

Note: Pupation rate = the number of pupation/the number of RNAi larvae; Aberration rate of pupae = the number of aberrational pupae/the number of pupation; Survival rate = the number of all surviving adults/the number of RNAi larvae; Aberration rate of adults = the number of aberrational adults/the number of surviving adults.

## Data Availability

The original contributions presented in this study are included in the article/Supplementary Materials. Further inquiries can be directed to the corresponding authors.

## Supplementary Materials

The following supporting information can be downloaded at: https://www.mdpi.com/article/10.3390/insects17060581/s1, Figure S1: Effects of *LsToll* silencing on the expression of Toll pathway-related genes in *L. sticticalis*.

## Appendix A

**Table A1:** Primers used in this study.

Primer Name	Sequence (5′ to 3′)	Title 3
*LsToll*-F	ATGGTAATGTACATCATATGGTCTCGT	RT-PCR
*LsToll*-R	TTAGTTTAGTAGGTCGCTATAACATTC	
si*LsToll-888*-F	GAGCUACAAUAGCAAUUUATT	siRNA synthesis
si*LsToll-888*-R	UAAAUUGCUAUUGUAGCUCT	
si*LsToll-946*-F	GGAGAUUCAACGUUUAAAUTT	
si*LsToll-946*-R	AUUUAAACGUUGAAUCUCCTT	
si*LsToll-1298*-F	CCGUCCCUAAAGAUAUAUUTT	
si*LsToll-1298*-R	AAUAUAUCUUUAGGGACGGTT	
si*LsToll-1695*-F	GCUUCUUCUAAGCUAUAAUTT	
si*LsToll-1695*-R	AUUAUAGCUUAGAAGAAGCTT	
si*NC*-F	UUCUCCGAACGUGUCACGUTT	
si*NC*-R	ACGUGACACGUUCGGAGAATT	
q*LsToll*-F	TGATTACAGCCGTCCCTAAAG	RT-qPCR
q*LsToll*-R	CTGGACTGAAGACGCCATCT	
q*LsMyD88*-F	CTTCATCCTCAGCATCTGGC	
q*LsMyD88*-R	TTGAGATGTCGAAGCGTAGG	
q*LsTube*-F	CAGGTTCAACAGACTCAGAG	
q*LsTube*-R	GACAGGCTAGTCAGAACCAA	
q*LsSad*-F	AACTGTTGGCAGCAGGGAG	
q*LsSad*-R	TGGCTCAGGCAAAATGTGCG	
q*LsSpo*-F	ACACGAGTCACCGTTCCAGG	
q*LsSpo*-R	AGGTCTTCCCCCGAAGAACT	
q*LsJHDK*-F	TCCACGTCTTCACGACGTTC	
q*LsJHDK*-R	GCATCAGCACCTTGGAGACC	
q*LsJHAMT*-F	AACGCGGAACTCTACCAGGG	
q*LsJHAMT*-R	GGCACATACTCCCGAAGAAGG	
q*LsJHEH*-F	CACCGAAGCAAGCTTGACG	
q*LsJHEH*-R	ACGACCCAAGTGGGAACTG	
