# Supplementary material for: LsToll Gene Mediates Antibacterial Immunity and Developmental Regulation in Loxostege sticticalis
Source: Insects. 2026 Jun 3;17(6):581. doi: 10.3390/insects17060581 (PMC13299791; doi:10.3390/insects17060581)
Supplement: Supplementary file 1 [file insects-17-00581-s001.zip › insects-4255290-supplementary.pdf]

Figure S1

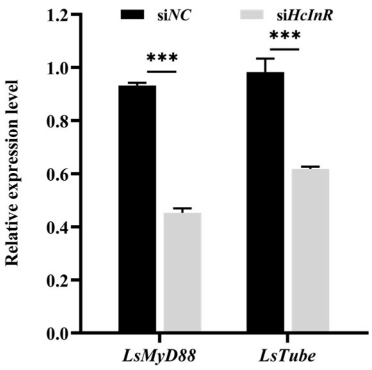

Figure S1. Effects of *LsToll* silencing on the expression of Toll pathway-related genes in *L. sticticalis*. Relative expression levels of *LsMyD88* and *LsTube* were determined by RT-qPCR after *LsToll* silencing. Data are expressed as mean ± SEM ($n = 3$). Significant differences were calculated using an unpaired Student's t-test (***, $p < 0.001$).
